# Incident gout and erectile dysfunction: is hyperuricaemia the elephant in the room?

**DOI:** 10.1186/s13075-017-1394-x

**Published:** 2017-08-10

**Authors:** Abhishek Abhishek, Michael Doherty

**Affiliations:** Academic Rheumatology, Division of Rheumatology, Orthopaedics, and Dermatology, School of Medicine, University of Nottingham, Nottingham, UK

**Keywords:** Gout, Erectile dysfunction

## Abstract

The first prospective population-based study to examine risk of erectile dysfunction in men with gout in the western world has been published. It reports that following their first diagnosis of gout, men have a 31% higher risk of erectile dysfunction than matched controls, although the absolute increase in risk is small. Of interest, the incidence of erectile dysfunction reported in this study is tenfold higher than those reported in nation-wide cohort studies from Taiwan. There is a need for further prospective cohort studies to examine the possible mechanistic association between gout, hyperuricaemia and erectile dysfunction.

The primary care-based prospective cohort study by Abdul Sultan et al. [1] using data from the UK’s Clinical Practice Research Datalink (CPRD) reports that following their first diagnosis of gout, men have a 31% higher risk of erectile dysfunction (ED) than matched controls, although the absolute increase in risk is small (e.g. 0.6% for a new consultation for ED, and 0.3% for receiving a prescription), reflecting a limited attributable effect on ED compared to other factors.

The authors are to be congratulated for undertaking this first prospective population-based study to examine risk of ED in men with gout in the western world. Only three other studies have examined ED in men with gout. One small hospital-based USA study surveyed people attending rheumatology clinics and reported that 76% of people with gout (n = 81) had ED compared to 51% of non-gout controls (n = 115) [2]. Interestingly, the incidence rate of ED reported in this CPRD study is tenfold higher than those reported in two nation-wide cohort studies using the National Health Insurance Research Database (NHIRD) in Taiwan [3, 4]. It is possible that this difference results from cultural reasons or lack of health insurance cover for ED in Taiwan [1]. However, hazard ratios for ED in those with gout in the Taiwan studies were comparable to the current study [1, 3, 4]. Given the prospective study design and source of recruitment we feel that this study provides reasonable estimates of rates of ED in men with gout in the western world [1], though the real prevalence is likely to be higher because many men may not consult their GP about it. Due to data limitations, the authors could not classify ED according to cause. However, data from the NHIRD showed a greater risk of incident organic ED than psychogenic ED in men with gout compared to men without gout (odds ratio (95% confidence interval) 1.52(1.03–2.22) vs. 1.18(1.03–1.35), respectively) [4], which suggests an important role for hyperuricaemia in causing vasculopathy and subsequent ED.

Abdul Sultan et al. [1] performed stratified and multivariate analyses to account for several confounders that may associate with gout and ED, and used landmark analysis to account for survivor bias. Interestingly, they found an elevated risk of ED prior to gout diagnosis, which suggests that ED may result from hyperuricaemia which precedes gout by some years [5]. However, the elevated risk for ED was restricted just to the 1-year period prior to gout diagnosis [1]. This suggests a possible delay between symptom onset and first consultation for gout, or that Read codes for gout are not applied at initial GP consultations, potentially due to diagnostic uncertainty in early stages of illness [6]. However, the control group for the analysis of association between prior diagnosis of ED and subsequent coding for gout was not selected according to the serum uric acid (SUA) and may have undetected hyperuricaemia, thereby minimizing the association. Interestingly, a previous CPRD study identified an increased risk of many comorbidities that may relate to chronic hyperuricaemia (including cardiovascular diseases and depression) in up to 10 years prior to initial gout diagnosis and a subsequent increased risk of the same comorbidities in the 5 years following diagnosis [7]. Depression was identified as an association with incident and subsequent gout in the current study and could have contributed to an increase in psychogenic ED. Thus, there is a need for further prospective cohort studies to examine the possible mechanistic association between hyperuricaemia and ED. Given that screening for hyperuricaemia is not standard clinical practice in the UK, such studies cannot be conducted using UK primary care data.

This study found no protective effect of urate lowering treatment (ULT) on the incidence of ED. This is not unexpected as most people with gout in the UK who receive ULT are on suboptimal doses and do not achieve target SUAs of <6 mg/dl [8]. Of interest, the authors found a trend towards higher risk of ED in those treated with ULT, although this was not statistically significant. This may be due to residual confounding by indication, as individuals with more severe disease and comorbidities are more likely to receive ULT. Such confounding could have been minimized by propensity score matching or adjustment. Thus, we feel that the lack of risk reduction for incident ED by ULT should not be interpreted as failure of ULT in preventing ED, since the suboptimal dosing of allopurinol in the UK is unlikely to exert any beneficial effect on vascular health, or indeed mortality [8, 9].

In summary, health care professionals should be aware that men with gout are at higher risk of ED, and might enquire about this during consultations. Further studies are required to determine whether the association between gout and ED is due to hyperuricaemia, or if gout confers a higher risk of ED than hyperuricaemia alone.

## Abbreviations

CPRD: Clinical Practice Research Datalink
ED: Erectile dysfunction
NHIRD: National Health Insurance Research Database
SUA: Serum uric acid
ULT: Urate lowering treatment
